# Prognostic value of preoperative von Willebrand factor plasma levels in patients with Glioblastoma

**DOI:** 10.1002/cam4.747

**Published:** 2016-05-28

**Authors:** Giovanni Marfia, Stefania Elena Navone, Claudia Fanizzi, Silvia Tabano, Chiara Pesenti, Loubna Abdel Hadi, Andrea Franzini, Manuela Caroli, Monica Miozzo, Laura Riboni, Paolo Rampini, Rolando Campanella

**Affiliations:** ^1^ Laboratory of Experimental Neurosurgery and Cell TherapyNeurosurgery UnitFondazione IRCCS Ca' GrandaOspedale Maggiore PoliclinicoUniversity of Milanvia F. Sforza, 35Milan20122Italy; ^2^ Division of PathologyDepartment of Pathophysiology and TransplantationFondazione IRCCS Ca' Granda Ospedale Maggiore PoliclinicoUniversity of Milanvia F. Sforza, 35Milan20122Italy; ^3^ Department of Medical Biotechnology and Translational MedicineLITA‐SegrateUniversity of Milanvia Fratelli Cervi, 93MI MilanSegrate20090Italy

**Keywords:** Angiogenesis, brain tumor, Glioblastoma, glioma., vascular endothelial growth factor, von Willebrand Factor

## Abstract

Circulating biomarker for malignant gliomas could improve both differential diagnosis and clinical management of brain tumor patients. Among all gliomas, glioblastoma (GBM) is considered the most hypervascularized tumor with activation of multiple proangiogenic signaling pathways that enhance tumor growth. To investigate whether preoperative antigen plasma level of von Willebrand Factor (VWF:Ag) might be possible marker for GBM onset, progression, and prognosis, we retrospectively examined 57 patients with histological diagnosis for GBM and 23 meningiomas (MNGs), benign intracranial expansive lesions, enrolled as controls. Blood samples were collected from all the patients before tumor resection. Plasma von Willebrand Factor (VWF):Ag levels were determined by using a latex particle‐enhanced immunoturbidimetric assay. The median levels of vWF:Ag were significantly higher in GBMs than in meningiomas (MNGs) (183 vs. 133 IU/dL,* P* = 0.01). The cumulative 1‐year survival was significantly shorter in patients with VWF:Ag levels >200 IU/dL than in those with levels <200 IU/dL and increased VWF levels were associated with a threefold higher risk of death in GBM patients. Our data suggest that VWF:Ag could be a circulating biomarker of disease malignancy, that could be considered, in association with other genetic and epigenetic factors, currently available in the GBM management. Future studies should investigate whether plasma VWF:Ag levels could also be used to monitor therapeutic effects and whether it may have a prognostic value.

## Introduction

Glioblastoma multiforme (GBM; World Health Organization, WHO, grade IV tumor) is the most malignant form of brain cancer with a median survival of 15 months 1. GBMs, are characterized by uncontrolled proliferation, diffuse infiltration of adjacent tissues, high genomic instability, and resistance to radio‐ and chemotherapy, all features that are useful prognostic factors for overall survival. Putative genetic and epigenetic prognostic factors have been investigated in order to optimize treatment strategies. These include 1p/19q codeletion, isocitrate dehydrogenase (IDH) 1 and 2 gene (*IDH1/2*) mutations, and methylation of the O(6)‐methylguanine‐DNA methyltransferase (MGMT) promoter 2, 3, which confer a better responsiveness to radio‐ and chemotherapy 4, 5, 6, even the disease is still incurable. A prominent feature of GBM is the aberrant angiogenesis. Indeed, GBM is considered the most hypervascularized tumor with activation of multiple proangiogenic signaling pathways that enhance tumor growth. In GBM patients, microvascular density has been correlated with postoperative survival 7. The identification of prognostic markers for GBM in more accessible specimens, such as blood, would be of help in the treatment of patients accompanied to the aforementioned genetic/epigenetic factors.

In the last years, new proteomic approaches allow the identification of circulating plasma proteins which reflect disease biology, and have been considered a useful option to discover potential biomarkers for brain tumors 8. Among the most relevant candidates, angiogenic factors, have been found to play a pivotal role in GBM onset and progression 9.

In this context, von Willebrand factor (VWF), a multimeric glycoprotein that carries coagulation factor VIII in plasma, participates in coagulation process, in hemostasis, and in negative modulation of angiogenesis 10. Besides these roles, VWF may also have proangiogenic properties. Yang and colleagues reported that higher mRNA and protein expression of VWF in gastric cancer (GC) tumor stroma may be regulated by the VEGF‐VEGFR2 signaling pathway in vitro and may contribute to GC progression in vivo 11. Moreover, Randi suggested that the mechanism of action of VWF in the control of angiogenesis involves enhanced signaling from the growth factor receptor VEGFR2. VWF was found to regulate two pathways, possibly linked, which may be controlling angiogenesis: an extracellular pathway involving integrin *α*v*β*3 and an intracellular pathway involving Ang‐2 storage in Weibel–Palade bodies (WPB). Both these pathways have been shown to influence VEGF signaling 10. Recently, VWF has been considered a potential circulating marker for tumor angiogenesis in different types of cancer 12. VWF is synthesized and released into the blood stream by endothelial cells, where it is also stored in the WPB from which it can be rapidly released upon stimulation 13. Several reports have described a direct interaction between VWF and tumor cells 14, 15, and a direct relationship between staining intensity of VWF in microvessels and different grades of astrocytomas 7.

The objective of this study was to evaluate the potential role of preoperative plasma levels of VWF in prognosis of GBM, in combination with the GBM molecular profile.

At this purpose, VWF antigen (VWF:Ag) plasma levels were evaluated in a cohort of patients affected with GBM and compared with a group of patients affected with meningiomas (MNGs), benign intracranial expansive lesions, in order to evaluate whether or not a correlation exists between VWF levels and disease prognosis.

## Materials and Methods

### Study population

A total of 57 patients with newly diagnosed GBM and 23 patients with MNGs, enrolled as controls, who had undergone neurosurgical procedures at the Neurosurgery Unit of the Fondazione IRCCs Ca' Granda Ospedale Policlinico in the last 3 years were included in the study. All tumors had been examined histopathologically and classified according to the WHO classification. Demographic and clinical data were retrospectively collected from patients' files, starting from the date of diagnosis until death or the date of the last visit performed, whichever came first. Karnofsky performance status (KPS) was assessed on the day before surgery, at which time, the collection of blood sample was performed. Written informed consent for blood sampling and molecular analyses was obtained from patients. The study was approved by the Institutional Review Board.

### Blood sample collection and VWF:Ag levels determination

Blood samples were taken from each subject prior to any treatment. Blood was drawn into tubes with sodium citrate anticoagulant and centrifuged and the supernatants were stored at −70°C until the levels of VWF:Ag were assessed. VWF:Ag levels were determined blind to the clinical data and were measured in plasma samples by using a latex particle‐enhanced immunoturbidimetric assay (Instrumental Laboratory, Orangeburg, NY) with a normal range of 40–169 IU/dL for O blood group and 55–165 IU/dL for non‐O blood groups.

### Molecular characterization of the tumor

Genomic DNA was extracted from peripheral blood lymphocytes (PBL) using the QIAamp DNA Mini Kit (Qiagen, Hilden, Germany) according to manufacturer's instructions. Tumor DNA was isolated from paraffin‐embedded tissue fragments using the Biostic FFPE tissue DNA isolation Kit (MO BIO Laboratories, Carlsbad, CA), following manufacturer's instructions.

Loss of heterozygosity (LOH) of chromosome arms 1p and 19q was assessed by short tandem repeats (STR: D1S468, D1S548, D1S2666, D1S1612, D19S918, D19S596, D19S206) analysis using paired tumor specimens and PBLs. Criteria used to establish LOH were the same as reported. 16. The *IDH1* codon 132 and *IDH2* codon 172 mutations were screened by MassArray iPLEX platform (Agena Bioscience, Hamburg, Germany) based on matrix‐assisted laser desorption ionization time‐of‐flight (MALDI‐TOF) mass spectrometry. PCR multiplex amplification was conducted in a 5 *μ*L reaction mixture containing 30 ng of tumor DNA, 100 nmol/L each designed primers, 100 nmol/L dNTP mix, PCR buffer, 25 mmol/L MgCl_2_, and 5U Taq DNA polymerase (Agena Bioscience). The mixture was incubated as follows: 95°C for 2 min, 45 cycles of 95°C for 30 sec, 56°C for 30 sec, 72°C for 60 sec, and then a final extension step at 72°C for 5 min. Plates were incubated at 37°C for 40 min and then at 85°C for 5 min. Finally, the iPLEX reaction mix, including iPLEX Buffer Plus, iPLEX Termination Mix, Extended Primers mix, and iPLEX enzyme (Agena Bioscience), was added to the PCR amplification products. The iPLEX reaction was carried out in the following conditions: at 94°C for 30 sec; 40 cycles at 94°C for 5 sec [52°C for 5 sec and 80°C for 5 sec (repeated five times per cycle)]; and a final extension step at 72°C for 3 min. The samples were spotted on a SpectroCHIP (Agena Bioscience). The chip was analyzed by mass spectrometry. The spectrum profiles generated by MALDI‐TOF‐MS were acquired and examined with SpectroTYPER 4.0 software (Sequenom, Inc., San Diego, CA). Moreover, tumor DNAs were subjected to conversion using the EZ DNA Methylation‐Gold Kit (Zymo Research Corporation, Irvine, CA). A total of 50–100 ng of DNA was amplified by PCR using 10 pmol of forward (5′‐GTTTYGGATATGTTGGGATAG‐3′) and reverse primers (5′‐bioCRACCCAAACACTCACCAAA‐3′). The PCR conditions for MGMT gene were: 95°C for 5 min, 45 cycles of 95°C for 30 sec, 56°C for 30 sec, and 72°C for 20 sec. Quantitative DNA methylation analysis of 10 CpGs (CpGs 74‐83) 17 was performed on the Pyro Mark ID instrument using the Pyro Gold Reagents and 1 pmol of sequencing primer (5′‐GATAGTTYGYGTTTTTAGAA‐3′).

### Tumor volume measurement

The tumor volume of each GBM patient was calculated from preoperative, post‐contrast T1‐weighted magnetic resonance images (MRI). The ellipsoid volume equation (AxBxC)/2, in which A, B, and C represent the three dimensions of the enhancing lesion detectable 18 was used. The MRI scans had been performed no more than 14 days before blood sampling.

### Statistical analysis

Continuous variables were expressed as median values and interquartile ranges (IQR), and compared by the Mann–Whitney *U*‐test. Categorical variables were expressed as frequencies and percent values, and compared by the chi‐squared test. Mortality rates with 95% confidence intervals (95% CI) were calculated as the number of events per 1 person‐year. Survival curves were plotted with the Kaplan–Meier method and analyzed by using the log‐rank test. For survival and regression analyses, VWF:Ag levels were categorized setting the cutoff level at the upper normal limit (165 IU/dL) and at supraphysiological levels (200 and 250 IU/dL). For the same purpose, the tumor volume was categorized by choosing as cutoff the median value observed in the study population (i.e., 25 cm^3^). Rho correlation coefficients were calculated by the Spearman's test. The Cox proportional hazard model was used to estimate potential risk factors of death and the effect of each variable was adjusted for the others in the multivariate model. In this model, death was the event and follow‐up duration was the time variable. The follow‐up period for each patient was calculated starting from the date of diagnosis to death or last visit at time of medical files review, whichever came first. Putative risk factors for reduced overall survival were VWF:Ag levels >200 IU/dL, age at surgery >60 years, preoperative KPS <70, tumor volume >25 cm^3^, methylated MGMT promoter, absence of 1p/19q deletion, and wild‐type *IDH1/2*. The results were expressed as crude and adjusted hazard ratios (cHR and aHR) with 95% CI. Starting with the full model, stepwise backward elimination was used as a variable selection procedure. A nominal *α* level of 0.10 as a cutoff for removing variables was considered. All *P*‐values reported are two‐sided and a value <0.05 was considered statistically significant. All analyses were done with SPSS software (release 21.0, IBM Corp., Armonk, New York).

## Results

### Characteristics of the study population

Overall, 80 patients who underwent neurosurgical procedures for GBMs or MNGs between February 2012 and June 2015 were included in the study. All patients underwent surgical resection of the lesion obtaining a gross total resection, except for two patients who underwent biopsy owing to tumor localization. All GBM patients received 6‐week treatment by radiotherapy (RT) with concomitant systemic therapy using alkylating agent, temozolomide (TMZ). In detail, standard treatment involved the administration total of 60 Gy in 30–35 fractions of 1.8–2.0 Gy, 5 days a week. Concomitant TMZ was administered 75 mg/m2/day during RT, and after 150‐200 mg/m2/day for 5 days every 28 days for 12 cycles 19. The main characteristics of the 80 patients at study entry are reported in Table 1. Blood group was known in 51 patients (64%), being O in 34 (43%). Since VWF tends to be lower in individuals with O blood groups 20, we analyzed separately VWF levels in both blood groups, and in spite of everything in GBM patients with O group, the levels were still higher respect to MNGs (median 250 IU/dL, IQR 175.50–355 vs. 164 IU/dL, IQR 121–200). Moreover, the same proportion of O/non‐O blood group was found analyzing separately patients with MNG and those with GBMs. Overall, the median preoperative KPS was 80 (IQR: 70–90) and it was inversely correlated with age (*r *= −0.43, *P* < 0.0001). In GBM patients, the median volume of the tumoral lesion was 25.6 cm^3^ (IQR: 8.0–45.3). The cumulative 1‐year survival was significantly shorter in patients with the bigger tumor volume at the end of the follow‐up period (median 32.5 vs. 23.4 cm^3^; IQR: 16.3–53.0 vs. 6.4–35.0, respectively; *P* = 0.04).

**Table 1:** Main characteristics of the 80 patients included in the study

	Patients with GBM (*n* = 57)	Patients with MNG (*n* = 23)
Male sex (%)	41 (62)	11 (48)
Median age at study entry, years (IQR)	61 (48–71)	66 (54–71)
Median follow‐up duration^a^, years (IQR)	0.9 (0.4–1.5)	2.5 (1.6–2.8)
Patients alive at the end of follow‐up (%)	21 (36)	23 (100)

IQR: interquartile range; GBM, glioblastoma; MNG, meningiomas.

a Follow‐up duration was calculated from the date of diagnosis to date of death or last follow‐up visit whichever came first.

### Plasma VWF:Ag levels in GBM and MNG patients

**Figure 1:**
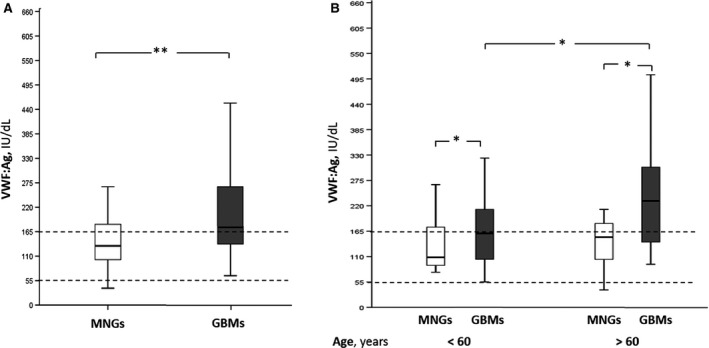
VWF:Ag plasma levels in MNG and GBM patients. (A) The box‐plots represent the interquartile range with median (central line); the whiskers represent the range of MNGs and GBMs measured in our patient cohort; (B) the box‐plot represent the VWF:Ag levels in MNGs and GBMs above 60 years of age, compared to younger. The dotted lines depict the interquartile ranges (IQR) of (VWF):Ag plasma levels in normal subjects. Mngs, Meningiomas. *P < 0.05; **P < 0.01. GBM, glioblastoma; MNG, meningiomas; VWF, von Willebrand Factor.

VWF:Ag levels were significantly higher in patients with GBM than in those with MNG (median 177 IU/dL, IQR: 135–294 vs. 133 IU/dL, IQR: 101–190, respectively; *P* = 0.01) (Fig. 1A). No differences were found by comparing VWF:Ag levels in patients with O versus non‐O blood group. VWF:Ag levels were linearly correlated with age both in patients with GBM (*r* = 0.40; *P* = 0.001) and in those with MNG (*r* = 0.45; *P* = 0.03). Moreover, when our cohort was divided into patients above 60 years and younger, we found that VWF:Ag levels were significantly higher in GBMs than in MNGs despite the age of patients (GBMs<60 years, median 158 IU/dL, IQR: 112–206; MNGs<60 years, median 109 IU/dL, IQR: 93–163; GBMs>60 years, median 231 IU/dL, IQR: 162–304; MNGs>60 years, median 143 IU/dL, IQR: 104–173, *P* < 0.05,) and furthermore, a statistically significant difference in VWF:Ag levels was observed in GBMs above 60 years of age, compared to younger (260 ± 145 IU/dL, vs. 196 ± 137 IU/dL, *P* < 0.05, Fig. 1B). In patients with GBMs, VWF:Ag levels were also linearly correlated with the tumor volume (*r* = 0.27; *P* = 0.04), while no correlation was found with the degree of MGMT promoter methylation.

### Survival analysis in patients with GBM

**Figure 2:**
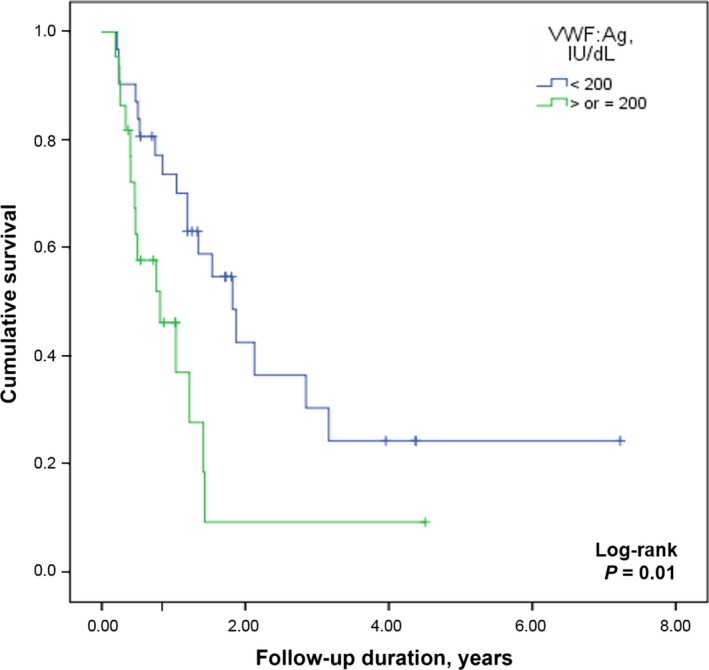
Survival rates among patients with GBM compared on the basis of VWF:Ag levels in plasma.Graph showing Kaplan–Meier estimates of overall survival in GBM patients who underwent gross total resection followed by standard‐of‐care radiation therapy and chemotherapy. The cumulative 1‐year survival in GBM patients with VWF:Ag levels >200 IU/dL (light gray line) was significantly shorter as compared with patients with VWF:Ag levels < 200 IU/dL (dark gray line) (43 vs. 73%; *P* = 0.009).GBM, glioblastoma; VWF, von Willebrand Factor

During the follow‐up, 34 patients (60%) died after a median of 0.8 years (IQR: 0.4–1.4) from diagnosis with a mortality rate of 0.4 per person‐year (95% CI 0.3–0.6). The main characteristics of this patient group are shown in Table 2. The genetic and epigenetic pattern of these patients was characterized by the presence of 1p/19q codeletion in two patients (3%), an unmethylated status of MGMT promoter in 28 (49%), and a wild‐type *IDH1/2* in 39 (68%). The cumulative 1‐year survival was significantly shorter in patients with vWF:Ag levels > 200 IU/dL (43 vs. 73% in patients with VWF:Ag < 200 IU/dL; *P* = 0.009; Fig. 2) and similar results were found comparing patients with VWF:Ag levels above or below 165 and 250 IU/dL (48 vs. 78% for both comparisons; *P* = 0.02 and *P* = 0.001, respectively) as reported for the whole study population. The cumulative 1‐year survival was shorter in patients with a tumor volume >25 cm^3^ (46 vs. 83% in those with tumor volume <25 cm^3^; *P* = 0.02). No difference in the survival rate was found by comparing patients with methylated and unmethylated MGMT promoter. By univariate Cox regression analysis, VWF:Ag levels >200 IU/dL (cHR 2.5; 95% CI: 1.2–5.1), age >60 years at time of surgery (cHR 2.6; 95% CI: 1.4–4.9), preoperative KPS <70 (cHR 2.5; 95% CI: 1.2–5.5), a tumor volume >25 cm^3^ (cHR 2.1; 95% CI: 1.1–4.2), and wild‐type *IDH1/2* (cHR 4.8; 95% CI: 1.3–16.9) resulted risk factors of decreased survival.

**Table 2:** Main characteristics of the 57 patients with GBM included in the study

Characteristic	
Median age at study entry, years (IQR)	62 (50–71)
Male sex (%)	36 (63)
Median VWF:Ag levels, IU/dL (IQR)	190 (141–294)
Median tumor volume, cm^3^ (IQR)	25.3 (8–46)
Median preoperative KPS, (IQR)	80 (70–90)
Median percentage of MGMT promoter methylation, (IQR)	8 (4–26)

IQR: interquartile range; VWF: von Willebrand factor; KPS: Karnofsky Performance Status; MGMT: O(6)‐methylguanine‐DNA methyltransferase; GBM, glioblastoma.

### Age‐matched survival analysis

Considering that VWF:Ag levels were strongly correlated with age, a subgroup analysis was performed including 23 patients with GBMs age‐matched with 23 patients with MNGs.

The median age was 66 years in both groups (IQR: 56–72 and 54–71 in GBMs and MNGs, respectively). VWF:Ag levels were still significantly higher in patients with GBM as compared with MNG patients (median: 133 vs. 237 IU/dL, IQR: 101–190 vs. 182–453, respectively; *P* < 0.0001). Also in this subgroup of patients, the 1‐year cumulative survival was significantly decreased in patients with VWF:Ag levels > 200 IU/dL (40 vs. 86% in those with VWF:Ag levels < 200 IU/dL; *P* = 0.001) and similar results were obtained by comparing patients with VWF:Ag levels above or below 165 or 250 IU/dL (61 vs. 84%, *P* = 0.03 and 33 vs. 84%, *P* = 0.0001, respectively). By univariate Cox regression analysis, VWF:Ag levels > 200 IU/dL were associated with reduced survival with a cHR of 5.8 (95% CI: 1.9–17.8).

## Discussion

Gliomas are the most common type of primary tumors of the central nervous system and among them, GBM is the most common primary malignant brain tumor in adults and with the worst prognosis 21. Several clinical predictors of survival have been identified, such as age, preoperative functional status, and tumor extent 22. In addition, some genetic and epigenetic features may be used as prognostic factors 2, 3. Nevertheless, despite many efforts to treat this disease, the mortality rate remains high, recurrence seems to be the rule, and still the outcome is invariably fatal. Malignant transformation results from the sequential accumulation of genetic alterations and abnormal regulation of growth factors signaling, including proangiogenic factors. GBMs are highly vascularized tumors and the process of angiogenesis is progressive throughout tumor development. Elevated levels of VEGF in serum/plasma of patients with gliomas seem to correlate with the microvascular density of the tumoral lesions 23 and current trials on GBMs with antiangiogenic agents, have shown a higher response rate and 6‐month progression free survival 24, but rather modest effects on overall survival 25. Plasma VWF, mainly deriving from endothelial cells, is often raised in patients with cancer and this may be due to an increased release in the neoplastic microenvironment where neoangiogenesis is a common feature. Furthermore, VWF is essential for WPB formation which contain several molecules involved in angiogenesis such as P‐selectin, angiopoietin‐2, and interleukin‐8 26. In addition, VWF is able to control VEGF signaling through multiple mechanisms involving both integrin *α*v*β*3 and angiopoietin 2 27, suggesting a role for VWF in angiogenesis modulation 10. In our study, patients with GBM had higher plasma VWF:Ag levels than those with benign expansive brain lesions. At variance with many of these, we took patients with benign brain expansive lesions as controls rather than healthy subjects considering that VWF:Ag usually raise in all patients with both benign and malignant neoplastic diseases 28, so rendering the finding of significantly higher levels in malignant gliomas more robust.

Increased VWF levels were associated with a threefold higher risk of death in patients with GBMs; however, since GBMs often occur in elderly individuals, the increase in VWF levels observed in this patient group could have been confounded by age, and indeed in the multivariate model, the risk associated with high VWF levels was no longer significant. For this reason, we performed a subanalysis in a group of age‐matched GBMs and MNGs, confirming that VWF levels were significantly related with survival irrespective of age. For the same reason, since VWF tend to be higher in individuals with non‐O blood groups 20, we checked that the distribution of ABO blood groups was similar between patients with GBMs and those with MNGs in order to rule out that the increased VWF levels were not attributable to a skewed blood group distribution. Indeed, as reported by Akin, differences in VWF levels between O and non‐O groups are more reliable for VWF values lower than 50 IU/dL, while in our patients, VWF levels are always higher, so as not to show significant differences between ABO groups 29.

In patients with GBM, survival was inversely related to VWF:Ag levels and tumor volume. However, MNGs that are high vascularized tumors have a benign course and much lower mortality rates 30. In a series of 28 patients with head and neck cancer, VWF levels were reported to be significantly higher in patients who died than in those who survived 31. In another series, VWF levels were significantly higher in 30 patients with malignant brain tumors as compared with 30 patients with benign brain tumors 32 but no regression analysis was performed to ascertain their prognostic value. All patients with GBM in our study had supraphysiological VWF:Ag levels and the higher was the VWF level the shorter was the survival rendering VWF:Ag levels a putative prognostic factor. Such a prognostic role for VWF has been shown in patients with other type of cancer, being high VWF levels associated with disease stage, 33, 34, 35, 36, 37, presence of metastases and/or lymph node status 33, 34, 36, 37, tumor size and residual disease after surgery 35. All in all, this study showed a significantly reduced survival in patients with GBMs and preoperative high levels of VWF:Ag in plasma irrespective of age. However, whether this finding is associated with the degree of angiogenesis within the neoplastic tissue or an abnormal or genetically deregulated pattern of VWF synthesis remains to be established by means of the experimental studies on GBM vascular compartment.

However, it should be emphasized that VWF is an acute phase inflammatory marker and it is, therefore, often difficult to establish exactly whether raised levels are causal or simply an epiphenomenon of the underlying cancer‐associated inflammatory process. For this reason, further studies are necessary to elucidate the aspects regarding the molecular pathways supporting the angiogenic role of VWF, although this possibility does not impinge upon our findings on the negative predictive values of this easily measurable plasma marker to better define prognosis and clinical outcome.

## Conflict of Interest

The authors declare no conflict of interest.
